# Ferroptosis-associated gene CISD2 suppresses colon cancer development by regulating tumor immune microenvironment

**DOI:** 10.7717/peerj.15476

**Published:** 2023-06-05

**Authors:** Yuanyuan Xu, Qingzhu Tang, Ning Ding, Tao Zhang, Hongbiao Luo

**Affiliations:** 1 Department of Anorectal Surgery, Chenzhou No. 1 People’s Hospital, Chenzhou, China; 2 Hunan University of Chinese Medicine, Hunan, China

**Keywords:** scRNA-seq, Ferroptosis, CISD2, Colon Adenocarcinoma (COAD), Tumor microenvironment homeostasis

## Abstract

**Background:**

Despite the association of ferroptosis with various tumors, the specific mechanism by which it influences colon adenocarcinoma (COAD) microenvironmental equilibrium remains elusive. This study aims to elucidate how ferroptosis affects COAD microenvironmental homeostasis and its potential impact on COAD research.

**Objective:**

By employing genetic screening and single-cell analysis of tumor data, we investigated the role of ferroptosis genes in COAD microenvironmental homeostasis. The genes were correlated with immune cell infiltration in tissue samples and patient outcomes.

**Methods:**

Ferroptosis-associated genes were initially identified through the FerrDb database. Utilizing the tidyverse and Seurat packages, genes with substantial expression differences were extracted, and clustering analysis was performed on the single-cell data. A Venn diagram depicted shared differential genes for ferroptosis and tumors. To screen key ferroptosis genes, further enrichment analysis and immune cell infiltration analysis were conducted. Lastly, human COAD cell lines were employed to overexpress CDGSH iron sulfur domain 2 (CISD2) through cellular assays to validate its function in COAD.

**Results:**

Following screening of The Cancer Genome Atlas (TCGA) and Genotype-Tissue Expression (GTEx) databases, 414 COAD patient samples and 341 normal samples were included. Through the FerrDb database, 259 ferroptosis genes were identified. Clustering the single-cell data revealed 911 tumor marker genes, of which 18 were ferroptosis genes. Analysis of variance (ANOVA) and univariate regression analysis determined that only CISD2 was statistically significantly associated with clinical outcomes. Additionally, CISD2 was found to positively correlate with activated memory T cells and negatively correlate with regulatory T cells (Tregs) and plasma cells in COAD, as well as being significantly associated with several immune-related and cancer-related pathways. CISD2 expression was elevated in most tumors, likely due to cell cycle regulation and immune system activation. Moreover, CISD2 upregulation inhibited COAD cell proliferation and enhanced 5-fluorouracil (5-FU) sensitivity. Our findings indicate, for the first time, that CISD2 governs the cell cycle and stimulates the immune system to impede COAD progression.

**Conclusion:**

By modulating the cell cycle and mediating immune infiltration, CISD2 may inhibit COAD development by influencing tumor immune microenvironment equilibrium, providing valuable insights into the relevance and potential impact of the research results on the COAD research field.

## Background

In 2020, colon cancer is projected to constitute approximately 6% of all cancer cases, with an expected 2.3 million new occurrences and nearly 1.8 million cancer-related fatalities (Sung et al., 2021). Colon cancer development is a heterogeneous malady stemming from the alteration of multiple pathways, culminating in homeostatic imbalance and subsequent tumor growth (Vogelstein et al., 1988). Due to the lack of suitable early diagnostic tools, roughly 20% of patients are diagnosed with metastatic colon cancer (Ciardiello et al., 2022). Although its pathogenesis remains enigmatic, colon cancer and its complications have deleterious impacts on individuals’ quality of life and life expectancy.

In recent years, substantial advancements have been made in the comprehension of molecular markers and the development of innovative treatment strategies for COAD (Lee & Chan, 2011; Nowak & Hornick, 2016; Punt, Koopman & Vermeulen, 2017; Gonzalez, Washington & Shi, 2017). These breakthroughs have led to a paradigm shift in the management of COAD, with an increasing emphasis on molecular profiling and personalized medicine (Amir-Aslani & Mangematin, 2010). These molecular markers have not only enhanced the prognostic accuracy but have also informed the development of targeted therapies. Some well-established emerging treatment strategies include immune checkpoint inhibitors, such as PD-1/PD-L1 and CTLA-4 blockers, as well as targeted therapies against EGFR, VEGF, and other signaling pathways (Ciardiello et al., 2006; Seidel, Otsuka & Kabashima, 2018; Rotte, 2019; Le et al., 2021). Furthermore, recent research has underscored the significance of ferroptosis and the tumor immune microenvironment in COAD (Xia et al., 2019). A deeper understanding of the interplay between ferroptosis and the immune microenvironment could pave the way for the development of groundbreaking therapeutic approaches, ultimately improving the prognosis for COAD patients.

Ferroptosis, a process necessitating metallic iron, phospholipids containing polyunsaturated fatty acid chains (PUFA-PLs), and reactive oxygen species (ROS), facilitates programmed cell death (Jiang, Stockwell & Conrad, 2021). Intricately connected to internal environmental homeostasis, ferroptosis partakes in diverse tumor progression stages and activates various tumor suppressors, such as p53 and BRCA1-associated protein 1 (BAP1) (Jiang et al., 2015; Zhang et al., 2018; Chen et al., 2021; Zhao, Yang & Yang, 2022). Oncogenes or oncogene signaling-mediated ferroptosis escape incites tumor initiation, progression, metastasis, and treatment resistance (Ubellacker et al., 2020; Yi et al., 2020). Accordingly, ferroptosis-related genes may play a major role in maintaining tumor microenvironment homeostasis. Consequently, ferroptosis-related genes likely play a pivotal role in maintaining tumor microenvironment equilibrium. Prostaglandin E2 (PGE2) impedes CD8+ T cell-mediated immune responses reliant on conventional type 1 dendritic cells (cDC1) and curtails cDC1 infiltration into tumor sites by inhibiting natural killer (NK) cell production of chemokine (C-C motif) ligand 5 (CCL5) and chemokine (C motif) ligand 1 (XCL1) (Zelenay et al., 2015; Böttcher et al., 2018). Ferroptosis has been associated with elevated prostaglandin-endoperoxide synthase 2 (PTGS2) expression and PGE2 release, promoting tumorigenesis when ferroptotic cells reach sufficient numbers (Yang et al., 2014, p. 4).

As malignant cells proliferate, they enlist adjacent nontransformed cells, ultimately forming the tumor microenvironment through the release of various intercellular communication factors, such as cytokines, chemokines, and vesicles (Balkwill, Capasso & Hagemann, 2012). Although metastasis is the primary cause of death in colon cancer patients, no universal mutation is culpable (Jones et al., 2008; Mlecnik et al., 2016). The malignant microenvironment, however, exhibits features like transforming growth factor (TGF)-β, which significantly augments the prevalence of cancer-associated fibroblasts (CAFs)-induced tumor-initiating cells. TGF-β signaling inhibitors can obstruct cross-signaling between cancer cells and the microenvironment, thereby arresting disease progression (Calon et al., 2015). Diminished type 1 T helper cell (TH1) activity or impaired immune cytotoxicity may also portend unfavorable outcomes in colon cancer patients (Galon et al., 2006; Mlecnik et al., 2016). Consequently, the tumor microenvironment, encompassing the immune microenvironment, might contribute more substantially to tumor prognosis than mutations alone.

Cancer immunotherapy, mainly employing immune checkpoint inhibitors and immune cell recombination therapy, has substantially improved patient survival outcomes, emphasizing the crucial significance of immunological and related biomarkers (Sharma & Allison, 2015; Hargadon, Johnson & Williams, 2018; Xu et al., 2021; Sun et al., 2022b; Mei, Li & Kang, 2022; Chen et al., 2022a; Luo et al., 2022). Nevertheless, immunotherapy’s efficacy remains relatively low in cancer patients. The tumor microenvironment comprises cancer cells and infiltrating immune cells, forming a complex and dynamic landscape. Tumor-infiltrating immune cells exhibit numerous connections with ferroptosis, a modality of cancer cell death. This study investigates the role of ferroptosis in maintaining the immune microenvironmental equilibrium in colon cancer. By exploring novel targets, colon cancer can be diagnosed and treated at earlier stages.

## Materials and Methods

### Data refinement and preprocessing

Single-cell sequencing information was obtained from the Gene Expression Omnibus (GEO) database, dataset GSE110009, comprising four specimens. In R language version 4.1.2, the tidyverse and Seurat packages were employed for single-cell RNA sequencing (scRNA-seq) data, and following batch effect removal, data integration and quality control were conducted. Additionally, The Cancer Genome Atlas (TCGA) provided sequencing and clinical information for colon adenocarcinoma (COAD) patients. The Genotype-Tissue Expression (GTEx) project supplied sequencing data for typical human colon tissues. TCGA and GTEx data were merged, and the limma package eliminated batch effects. Gene expression data normalization and scaling were carried out using the Trimmed Mean of M-values (TMM) method and log2 transformation. The FerrDb database offered information on ferroptosis genes. Factors such as patient age, sex, and tumor stage were considered in the analysis as potential confounders. A multivariate regression model was used to adjust these factors, assessing the independent association between the identified biomarkers and outcomes of interest. To further support quality control and preprocessing steps, comparisons of data distribution and quality metrics before and after preprocessing were performed. This comparison aimed to demonstrate the effectiveness of preprocessing methods in removing technical biases and preserving biologically relevant information.

### Examination of scRNA-seq data

Quality control of the GSE110009 single-cell data was conducted, excluding samples with less than 200 detected genes and mitochondrial gene content exceeding 5%. Furthermore, cells with extreme quantities of unique molecular identifiers (UMIs) were omitted. We extracted 2,000 highly variable genes between cells for further analysis. Data normalization was executed using the NormalizeData function, allowing observation of each component’s contribution to features. Dimensionality reduction was performed using Principal Component Analysis (PCA), retaining the top 20 principal components for downstream analysis. Cell clustering was carried out using the t-distributed stochastic neighbor embedding (tSNE) algorithm. Marker genes for each cluster were identified using the FindAllMarkers function, with a minimum log-fold change of 0.25 and a False Discovery Rate (FDR) threshold of 0.05. Heatmaps were generated based on the top 10 marker genes for each cluster. Subsequently, cell types were annotated for each cell cluster and manually verified and corrected using the CellMarker website as a reference (Zhang et al., 2019).

### Identification of ferroptosis-colon cancer associated genes and construction of protein-protein interaction (PPI) network

Ferroptosis genes were overlapped with colon cancer genes. Pearson correlation analysis was performed to determine the correlations between intersecting genes with a correlation coefficient threshold of |r| > 0.3 and a *p*-value < 0.05. The PPI network of intersected genes was visualized using STRING and Cytoscape, with a combined score threshold of 0.4.

### Enrichment analysis

Co-associated genes between ferroptosis and colon cancer were subjected to enrichment analysis using the Kyoto Encyclopedia of Genes and Genomes (KEGG) and Gene Ontology (GO) databases as pervious studies (Chen et al., 2017; Wu et al., 2021; Sun et al., 2022a). These databases were selected based on their comprehensive and well-curated annotations for biological processes, molecular functions, cellular components (GO), and metabolic and signaling pathways (KEGG). We converted gene names to ENTREZID using the R package org.Hs.eg.db. For enrichment analysis, we employed the R package clusterProfiler (Wu et al., 2021), which uses hypergeometric tests to determine the statistical significance of gene set overlaps. Multiple testing corrections were performed using the Benjamini-Hochberg method to control the false discovery rate (FDR). Enriched KEGG pathways and GO terms were considered significant at an FDR-adjusted *p*-value threshold of 0.05. The visualization of enrichment analysis results was carried out with the ggplot2 package, generating bar plots and dot plots to represent the enriched terms and pathways, along with their corresponding gene ratios and adjusted *p*-values. To further support the enrichment analysis gene set enrichment analysis (GSEA) was performed as previous research (Subramanian et al., 2007; Powers et al., 2018; Chen et al., 2022b).

### Differential expression and prognostic analysis

To ascertain the differential expression of ferroptosis-colon cancer co-associated genes, two independent sample rank sum tests were employed. Univariate Cox regression analysis evaluated genes associated with colon cancer to identify gene prognostic indicators.

### Immune microenvironment

The CIBERSORT website compared 22 immune cell infiltrations between colon cancer and normal samples, as well as 46 prevalent immune checkpoints (Chen et al., 2018). The TIMER website verified the relevance of key genes to immune cells (Li et al., 2017).

### Single gene analysis

Genes were classified into high and low expression groups based on their median expression. Receiver operating characteristic (ROC) curves determined key genes’ diagnostic efficacy. Kaplan-Meier (KM) curves evaluated the significance of key genes for the prognosis of colon cancer patients.

### Construction of PPIs for key genes and enrichment and differential analysis

The GeneMANIA website searched for genes interacting with key genes (Franz et al., 2018). STRING sought protein interactions between key genes, and Cytoscape visualized the data (Szklarczyk et al., 2021). Gene Set Variation Analysis (GSVA) enrichment analysis identified differential pathways between high and low expression groups of key genes (Hänzelmann, Castelo & Guinney, 2013). The clusterProfiler package conducted Gene Set Enrichment Analysis (GSEA) (Yu et al., 2012). The TCGA and GTEx databases were integrated for key gene differential analysis in pan-cancer.

### Copy number variations in ferroptosis key genes in cancer

The TCGA database provided data on copy number variations (CNVs), methylation, and mutations. Ferroptosis key genes were categorized as activator genes, suppressor genes, and unclassified. The randomcoloR package was employed to analyze CNV variants of ferroptosis key genes in cancer. To examine the expression profiles and CNV correlations of ferroptosis key genes, Spearman correlation analysis was conducted. The chAMP package was utilized to analyze methylation differences between ferroptosis key genes in tumor and normal tissues (Ramey et al., 2019). To investigate the relationship between ferroptosis key gene expression profiles and gene methylation, Spearman correlation analysis was employed. The Magrittr package was used to assess the frequency of mutations in ferroptosis key genes in cancer.

### Examination of crucial ferroptosis genes in colorectal carcinoma

GEPIA2 was employed to analyze survival in relation to ferroptosis-colorectal carcinoma co-associated genes (Tang et al., 2019), plotting KM survival curves for high and low median expression groupings of key genes.

### GSEA enrichment analysis of vital genes at the pan-carcinoma level

Twenty cancers were classified as high or low expression based on the median expression of essential genes. To explore the role of these genes in cancer, we investigated the enrichment pathways of differentially expressed genes between groups using clusterProfiler (Yu et al., 2012).

### Cellular experiments

Human colorectal carcinoma cell lines HCT116 were acquired from the Shanghai Cell Resource Center of the Chinese Academy of Sciences. Cells were cultured in DMEM complete medium supplemented with 10% fetal bovine serum and 1% penicillin-streptomycin at 37 °C in a humidified incubator with 5% CO_2_. Cells were seeded at a density of 1 × 10^5^ cells/mL in 6-well plates for all experiments. Sigma, Poole, UK, supplied 5-FU (F6627-10G) dissolved in dimethylsulfoxide. Working concentrations of 5-FU were prepared by diluting the stock in complete culture medium immediately before treatment. Cells were treated with 100 µM 5-FU or equivalent volumes of DMSO (vehicle control) for 48 h after 24 h of initial seeding. Treatment groups were established: control, CISD2-OE, control+5-FU, CISD2-OE+5-FU, shCISD2-1, and shCISD2-2. After the drug treatment, scratch assays were performed to assess cell migration. Scar formation was examined under an inverted microscope at 0 and 30 h post-scratch. ImageJ was used to measure cell scratch distances, and the migration rates of each cell group were calculated using the formula (30 h edge distance − 0 h edge distance)/0 h edge distance × 100%.

### Stabilization of transfected cell lines

To construct the CISD2 vector, PCR-amplified human CISD2 cDNA was subcloned into pCDHCMV-MCS-EF1-copGFP and pCDH-CMV-MCS-EF1-puro (System Biosciences, Palo Alto, CA, USA), as described in prior research. Following the manufacturer’s instructions, the empty vector or lentiviral recombinant vector was co-transfected into HEK-293T cells along with packaging plasmids (pMD2.G and psPAX2); target cells were infected by lentiviral supernatants. Lipofectamine3000 (Invitrogen, Carlsbad, CA, USA) was employed to transfect HCT116 cells, which exhibit low endogenous CISD2 expression. Puromycin (Invitrogen, Carlsbad, CA, USA) was utilized to select antibiotic-resistant HCT116 cells, and western blotting verified all transfections.

### Constructing expression vectors

Previously reported target shRNA sequences were subcloned from pLVX-shRNA (Takara) to suppress CISD2 (Shao et al., 2021). Recombinant lentiviral plasmids were purified using an extraction kit (Accurate Biotechnology, Hunan, China) and verified *via* sequencing. To ascertain the vector’s efficacy, it was combined with a vector containing scrambled DNA. Lentiviruses were packaged using 293T cells, with pLVX-shRNA, pMD2G, and pSPAX2 lentiviral plasmids co-transfected into 293T cells. Recombinant lentiviruses for silencing CISD2 were generated using a lip3000 transfection kit (Shao et al., 2021).

### CISD2 suppression

HCT116 cells were transfected with scramble or CISD2 knockdown lentivirus using six-well plates (NEST Biotechnology) at an MOI of 20, as reported earlier (Shao et al., 2021). After 24 h of infection, cells were harvested *via* centrifugation, resuspended in preheated complete culture medium, and administered selective antibiotics. Following a 7-day screening, CISD2 expression was gauged through immunoblotting. Verified cells were then used for subsequent experimentation.

### Western blot

Cells were cleansed twice with phosphate-buffered saline (PBS). Subsequently, the total protein extracts were procured by lysing the cells in chilled RIPA lysis buffer (P0013B; Beyotime, Jiangsu, China) for a quarter of an hour, followed by centrifugation. To execute electrophoresis and membrane transfer, 20 g samples were loaded into each lane of the SDS-PAGE gel. The primary antibody was incubated overnight at 4 °C. Post incubation, the membrane was cleansed with tris-buffered saline containing 0.1% Tween-20 (TBST) and exposed to the secondary antibody for an hour. Visualization was achieved through chemiluminescence.

#### Quantitative real-time polymerase chain reaction (qRT-PCR)

Total RNA was extracted from HCT116 cells utilizing Tiangen’s TRNzol-A+ reagent (Beijing, China). The qRT-PCR analysis was performed using total RNA and primers as previously described (Huang et al., 2015). Standard curves facilitated the determination of relative RNA levels in each specimen. To ascertain the integrity of the cDNA input, β-actin served as an internal control.

### Cell viability assay

The viability of the reconstituted cells seeded into 96-well culture plates was assessed employing the Cell Counting Kit-8 (CCK8) assay kit (Meilunbio, Dalian, China). The reagent generates a soluble yellow formazan product in the cell culture medium by reducing WST-8 through cellular dehydrogenases, exhibiting a distinct absorption peak at 450 nm.

### Statistical analysis

For contrasting pairs of groups, the Student’s t-test was employed, while for multiple group comparisons, one- or two-way ANOVA with Tukey’s multiple comparison test was utilized. Statistical significance was defined as *p* < 0.05.

## Results

### Ferroptosis and colon cancer associations uncovered through single-cell analysis

**Figure 1:**
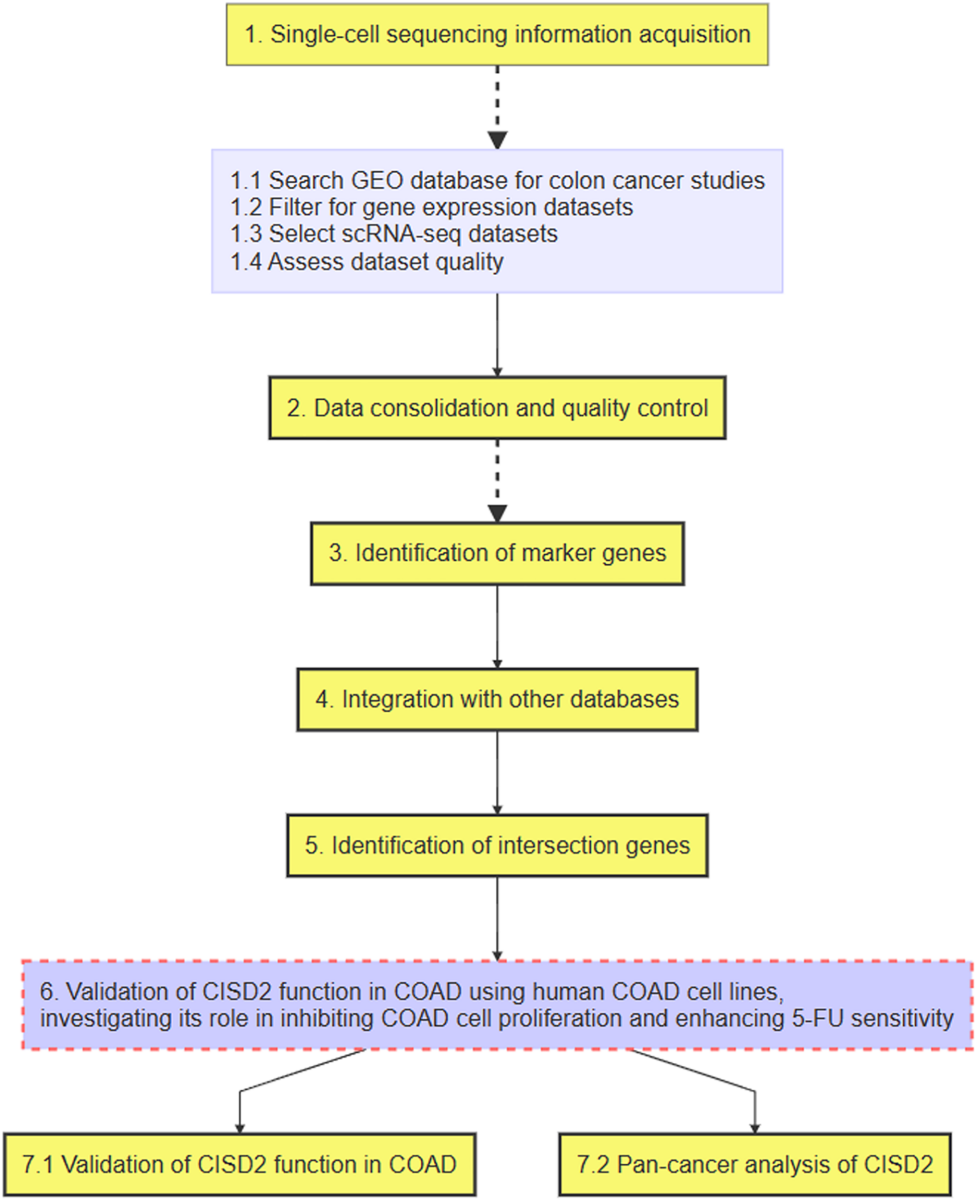
Flowchart of the research process.

**Figure 2:**
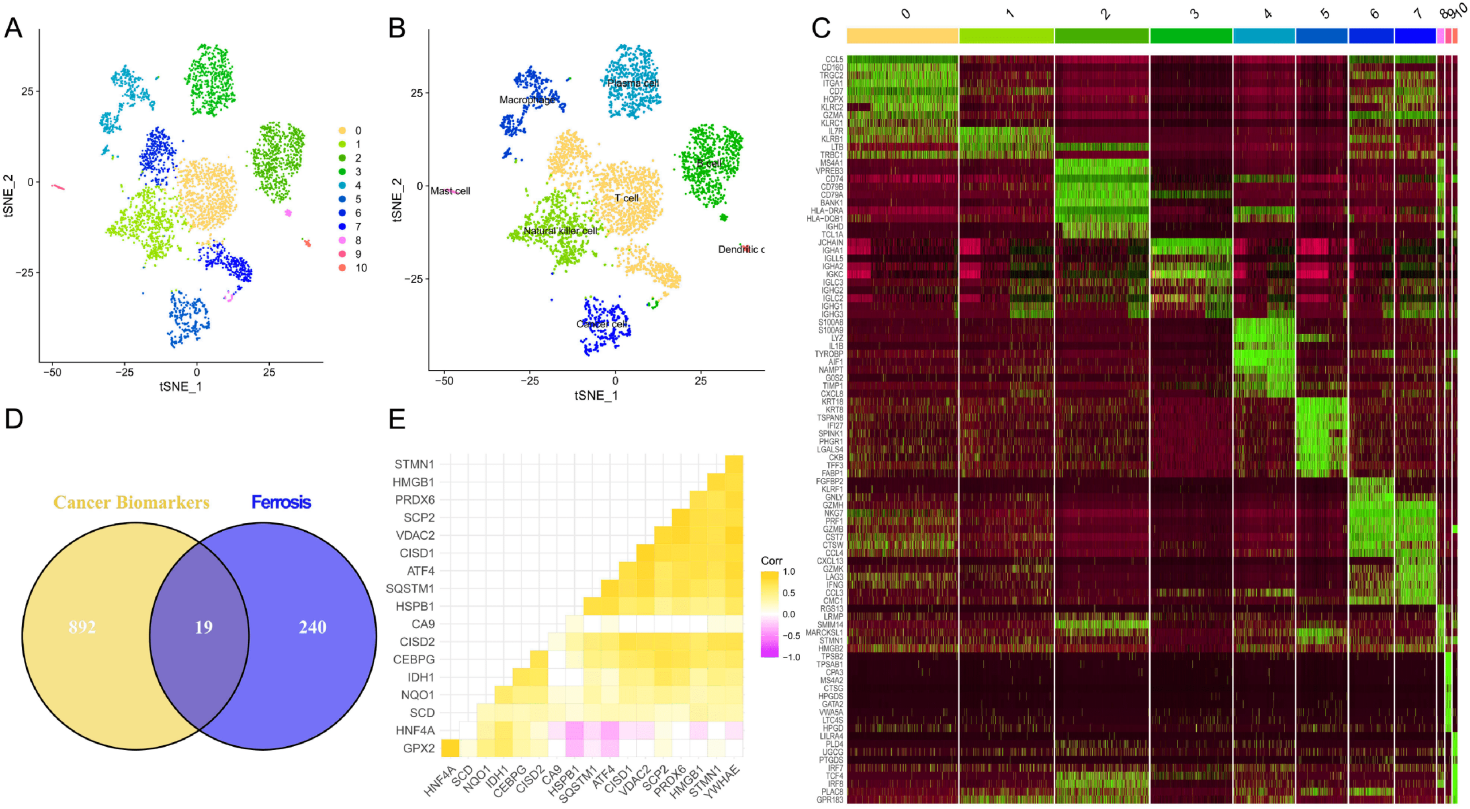
Biomarkers correlated with tumor ferroptosis in single cells. (A) Dimensionality reduction of single-cell data *via* t-Distributed Stochastic Neighbor Embedding (tSNE), clustered into 11 categories; (B) cellular annotation, classified into a total of eight groups; (C) top 10 differentially expressed genes for the 11 cell types; (D) Venn diagram depicting the overlap of tumor biomarkers and ferroptosis genes; (E) correlation heatmap illustrating the association of 18 ferroptosis genes.

The flowchart of this study is shown in Fig. 1. Upon integrating four samples from the GSE110009 dataset through cell clustering (Fig. 2A), cell annotation revealed eight distinct subgroups: mast cells, natural killer cells, T-cells, macrophages, plasma-cells, B-cells, dendritic-cells, and cancer-cells (Figs. 2B and 2C). Among 911 colon cancer marker genes, 19 were found to be associated with ferroptosis (Fig. 2D). Further analysis of the 18 ferroptosis-colon cancer co-associated genes, excluding ATP5MC3, revealed significant correlations (Fig. 2E) and protein-protein interactions (Fig. S1B).

### Exploration of ferroptosis-related genes

**Figure 3:**
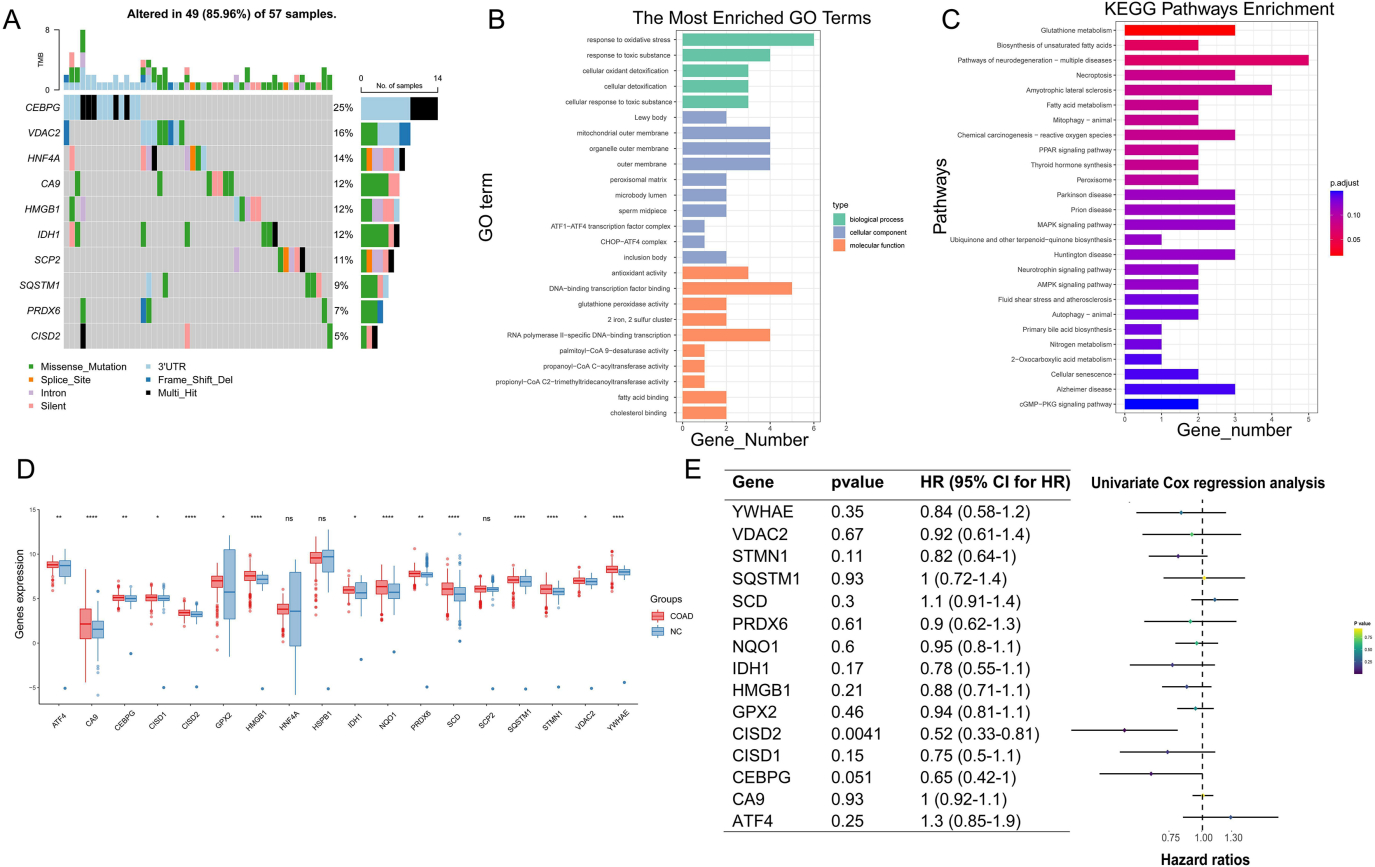
Examination of crucial genes in ferroptosis. (A) Waterfall chart displaying the mutational analysis of 18 genes. (B) Bar diagram representing Gene Ontology (GO) enrichment results. (C) Bar diagram showcasing Kyoto Encyclopedia of Genes and Genomes (KEGG) enrichment results. (D) Box plot illustrating the differences of 18 genes between normal and Colon Adenocarcinoma (COAD) specimens. (E) Univariate regression analysis of 15 differing genes. In Figure, ‘ns’ denotes a result that is not statistically significant, while asterisks (*, ** and ****) represent significance levels of 0.05, 0.01, and 0.0001, respectively.

Following the integration of TCGA and GTEx data and the removal of batch effects (Figs. S1C and S1D), PCA validation confirmed data integration (Figs. S1E and S1F). In COAD pathogenesis, CEBPG, VDAC2, and HNF4A carried high mutational loads and potentially played crucial roles (Fig. 3A). GO enrichment analysis suggested significant associations between ferroptosis-colon cancer co-related gene functions and pathways such as oxidative stress response, toxic substance response, and DNA-binding transcription factor binding (Fig. 3B). Additionally, KEGG enrichment analysis highlighted Glutathione metabolism and Biosynthesis of unsaturated fatty acids pathways (Fig. 3C). Notably, only three genes were statistically significant in the Ferroptosis-Colon Cancer Common Associated Gene Difference Analysis (Fig. 3D). The remaining 15 ferroptosis genes were suggested to play important roles in COAD, and a one-way COX regression analysis showed that only CISD2 had a statistically significant impact on prognosis (Fig. 3E).

### Immune microenvironment examination

**Figure 4:**
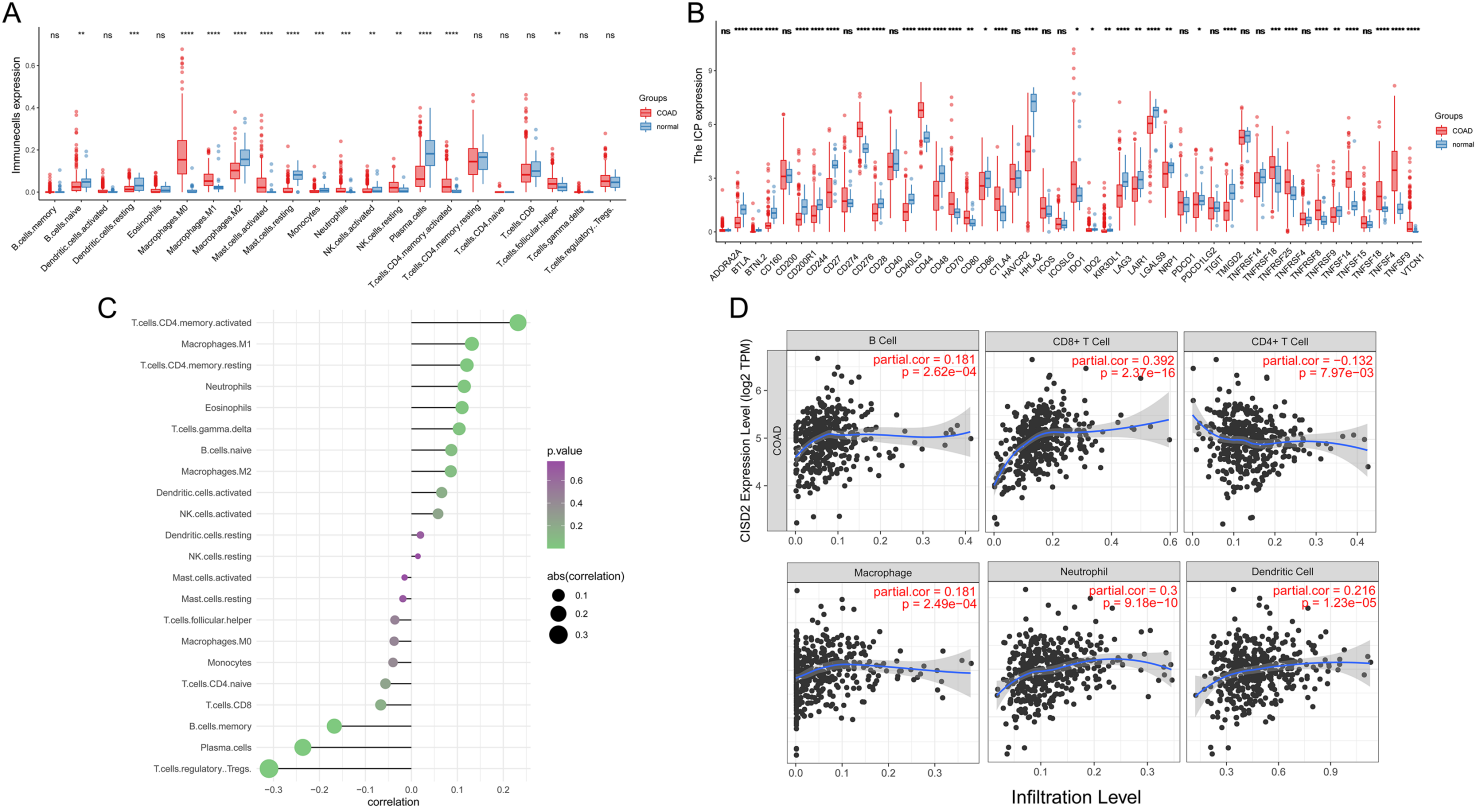
Immune microenvironment assessment. (A) Box plots exhibiting disparities in 22 immune cells between tumor and healthy samples. (B) Box plots revealing variations in immune checkpoints between tumor and normal samples. (C) Association between 22 immune cells and CISD2. (D) Correlation of CISD2 and six immune cells in colorectal cancer. In Figure, ‘ns’ denotes a result that is not statistically significant, while asterisks (*, **, *** and ****) represent significance levels of 0.05, 0.01, 0.001, and 0.0001, respectively.

In colorectal cancer samples, macrophages M0 and M1 displayed greater infiltration compared to normal samples, aligning with general findings (Fig. 4A). Colorectal cancer samples also exhibited increased infiltration of mast cells activated, neutrophils, NK cell resting, T cells CD4 memory activated, and T cells follicular helper. Conversely, CD8 T cells and NK cells activated, which are more effective in killing cancer cells, demonstrated reduced infiltration in colon cancer, potentially contributing to progression and poor prognosis. Colon cancer samples also expressed more immune checkpoints, possibly leading to increased immune escape (Fig. 4B). Cibersort results revealed that CISD2 positively correlated with T cells memory activated (*p* < 0.001) and negatively correlated with T cells regulatory Tregs and Plasma cells (*p* < 0.001) (Fig. 4C). CISD2 also showed positive correlations with B cells, neutrophils, macrophages, CD8 T cells, and dendritic cells (*p* < 0.05) (Fig. 4D), indicating a significant relationship between CISD2 and immune response.

### Discrepancies in CISD2 expression

**Figure 5:**
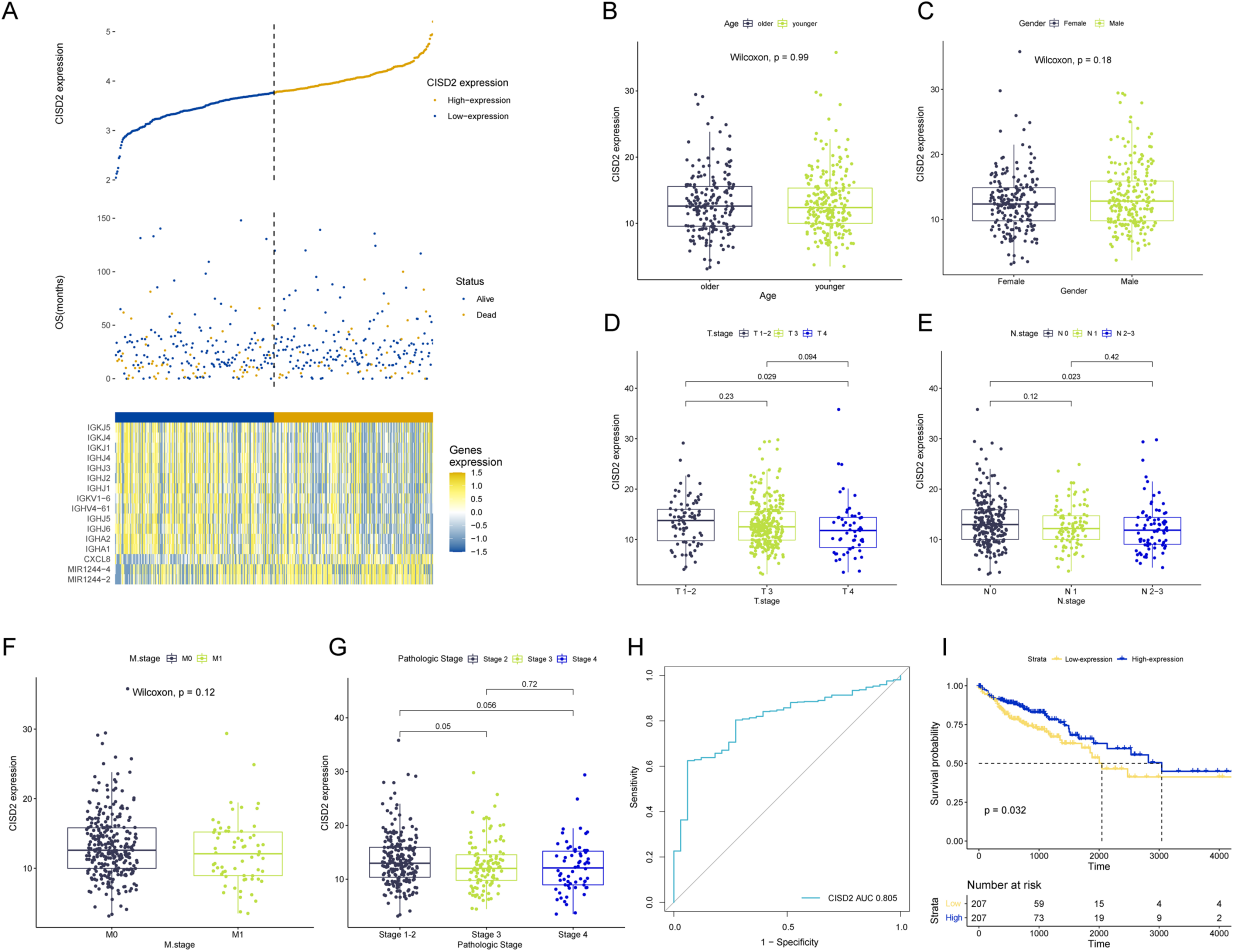
Analysis of CISD2. (A) Survival data for high and low CISD2 subgroups and display of diûerential genes. (B–G) Association between patient age, sex, T-stage, N-stage, M-stage, and overall stage with CISD2 expression. (H) Receiver Operating Characteristic (ROC) curves demonstrating the diagnostic accuracy of CISD2. (I) Survival curves for discerning prognostic disparities between groups with and without CISD2 expression.

CISD2 expression levels reveal notable distinctions between high and low expression TCGA colon cancer samples, examined through comparative analysis (Fig. 5A). Clinical stratification demonstrates a higher prevalence of CISD2 at advanced T-stages and initial N-stages (*p* < 0.05) (Figs. 5B–5G). The ROC curve suggests superior diagnostic efficacy of CISD2 over CISD1 (Fig. 5H). Consequently, CISD2 is considered a protective factor for colon cancer, exhibiting a robust immunological connection. CISD2 exhibits a strong association with CISD1 and CISD3, as illustrated by the gene interaction network (Fig. S2A). PPI identifies proteins interacting with CISD2, potentially influencing its expression and function (Fig. S2B). Elevated CISD2 expression correlates with increased protein secretion, MTORC1 signaling activation, and E2F target activation. Thus, high CISD2 expression is intimately linked to cell cycle regulation and growth metabolism. Conversely, low CISD2 expression displays increased KARS signaling DN, hedgehog signaling, myogenesis, and apical surface activation, suggesting interactions with colon cancer-related pathways (Fig. S2C). This insinuates a strong immune response association for CISD2, potentially impeding colon cancer progression (Fig. S2D). In a pan-cancer analysis, CISD2 was highly expressed in most colon tumors (*p* < 0.001) (Fig. S2E).

### CISD2 upregulation impedes proliferation and tumor-promoting activity in colon cancer cells, enhancing 5-FU sensitivity

**Figure 6:**
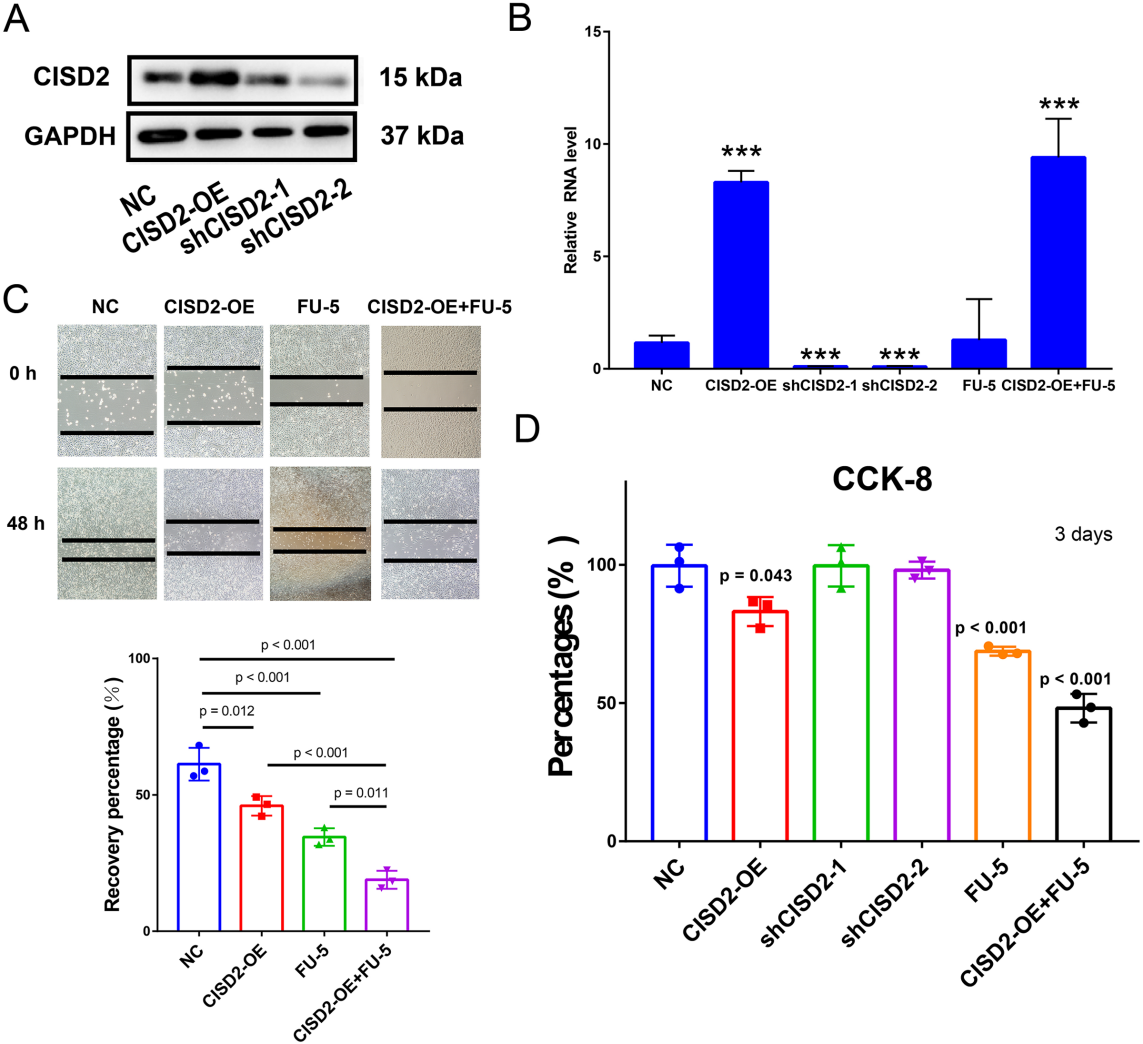
Proliferation, invasion, and migration of HCT116 cells linked to CISD2. (A,B) Validation of CISD2 relative expression by western blot (A) and Quantitative Reverse Transcription Polymerase Chain Reaction (qRT-PCR) (B), <*** = indicates *p* < 0.001; (C) wound-healing assays employed to evaluate the impact of CISD2 knockdown on cell migration; (D) HCT116 cells treated with 5-Fluorour ouracil (5-FU) (5 mM). The Cell Counting Kit-8 (CCK8) assay was utilized to measure cell viability, normalized to cells in the presence of Dimethyl Sulfoxide (DMSO) (vector control) set to 100% for each cell line. The data represents the mean ± Standard Deviation (SD) of three independent experiments (*n* = 3).

To explore CISD2’s potential role in colon cancer cell proliferation and carcinogenesis, we established HCT116 cell lines stably expressing CISD2. Western blots detected CISD2 protein expression levels in transfected and control cells. CISD2 overexpression upregulated protein levels, while shCISD2-1 and shCISD2-2 downregulated them (Fig. 6A). OD-CISD2 cells exhibited significantly elevated CISD2 mRNA levels compared to shCISD2 transfected cells, as determined by PCR (*p* < 0.001, t-test) (Fig. 6B). Assessing CISD2’s impact on colon cancer cell invasiveness, we conducted a wound healing assay. Control cells nearly healed 48 h post-scratch creation, while CISD2 cells did not. Overexpression CISD2 groups displayed fewer healing areas compared to control groups (*p* = 0.012, t-test) (Fig. 6C). The invasive propensity of colon cancer cells is curtailed by the augmentation of CISD2 expression. The CCK-8 assay demonstrated a notable reduction in the HCT116 cell count overexpressing CISD2 (*p* = 0.043 *vs* NC, t-test) (Fig. 6D). An empty vector-transfected control group experienced increased cell growth and proliferation compared to CISD2 overexpressing cells. Notwithstanding the application of FU-5, CISD2 expression persisted in overexpressed cells (*p* < 0.001, t-test) and remained unaltered by FU-5. Figure 5C depicts the inhibition of scratch healing in CISD2 cells by FU-5 (*p* < 0.001, t-test). Additionally, Fig. 5C reveals a considerable enhancement of scratch healing following CISD2 overexpression (*p* = 0.011, t-test). HCT116 cells with CISD2 overexpression were also substantially reduced by FU-5 after the CCK-8 viability assay (*p* < 0.001, t-test) (Fig. 6D). Consequently, the upregulation of CISD2 may bolster the sensitivity of HCT116 cells to FU-5 treatment.

#### Oncogenic genetic variations in ferroptosis-associated genes

**Figure 7:**
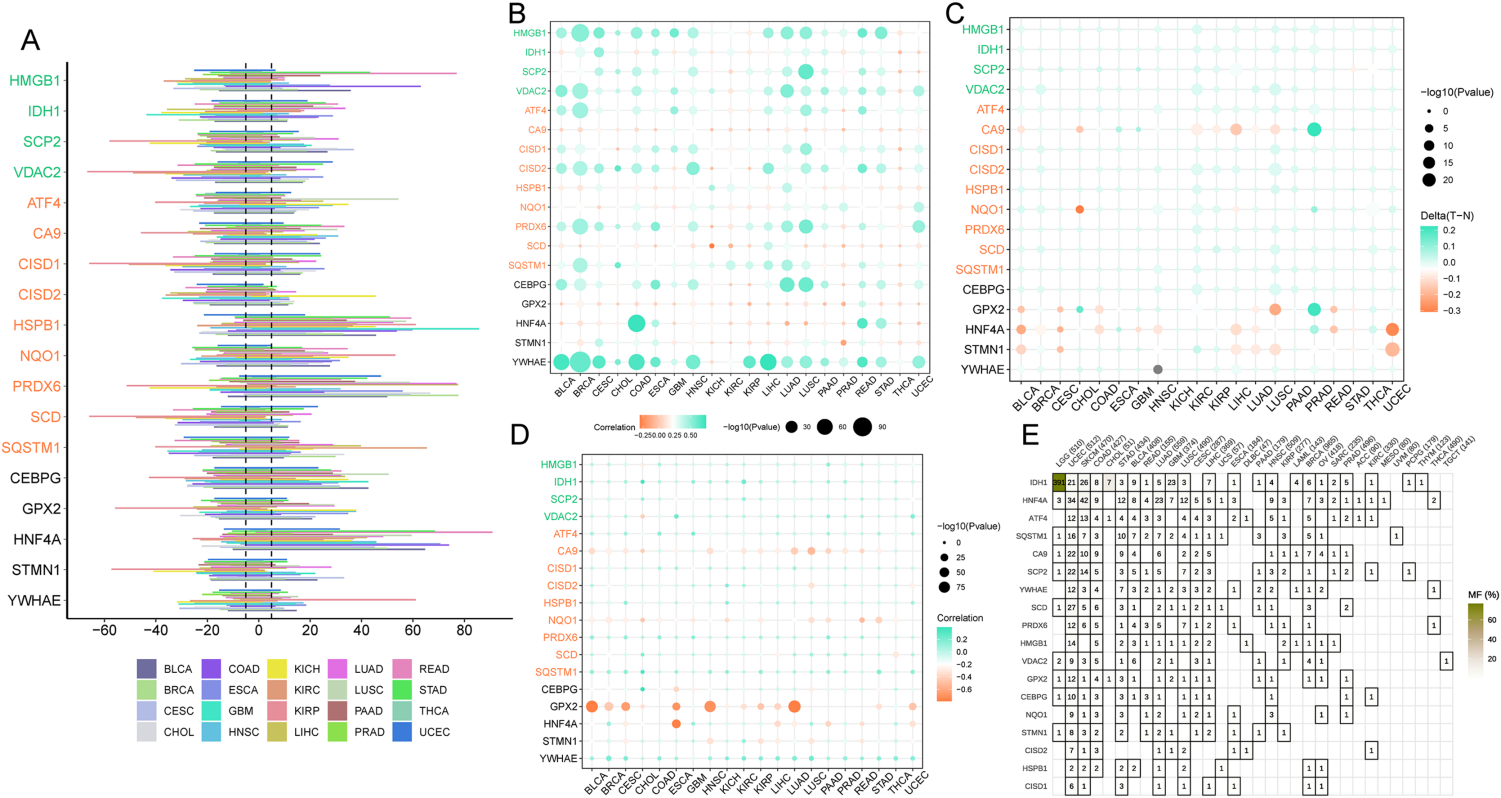
Copy Number Variation (CNV), methylation, and gene mutations in pan-cancer. (A) Expression of ferroptosis genes in pan-cancer. (B) Dot plot illustrating the correlation between gene expression and CNV of ferroptosis genes. (C) Comparison of ferroptosis gene methylation between tumors and normal samples. (D) Relationship between ferroptosis gene expression and gene methylation. (E) Tumors containing mutations in the ferroptosis gene.

Ferroptosis genes SCD, CISD1, and CISD2 displayed a greater predisposition to somatic copy number deletions compared to HSPB1, HNF4A, and PRDX6 (Fig. 7A). The correlation between somatic copy number mutations and gene expression in tumors was investigated, as these mutations govern gene expression in neoplasms. The majority of tumors exhibited significant somatic copy number mutations linked to ferroptosis genes (Fig. 7B). These findings imply that copy number aberrations in ferroptosis genes are prevalent in most cancers and can influence gene expression. In most cancers, GPX2, HNF4A, and STMN1 were hypomethylated (Fig. 7C), while the majority of cancer genes exhibited hypermethylation (Fig. 7A). Promoter DNA methylation in most cancers was observed for ferroptosis-cancer co-associated genes based on this outcome. It is plausible that these co-associated genes are less regulated by methylation (Fig. 7D) due to the correlation analysis of methylation levels and gene expression. In contrast, GPX2, CA9, NQO1, and HNF4A displayed the opposite effect. Thus, these findings suggest that methylation may play a more significant role in regulating GPX2, CA9, and HNF4A gene expression than somatic copy number mutations. Mutation analysis revealed elevated mutation rates for IDH1, HNF4A, and ATF4 in LGG, UCEC, and SKCM, as well as higher mutation rates for this ferroptosis-cancer co-associated gene.

#### Pan-cancer immune microenvironment

**Figure 8:**
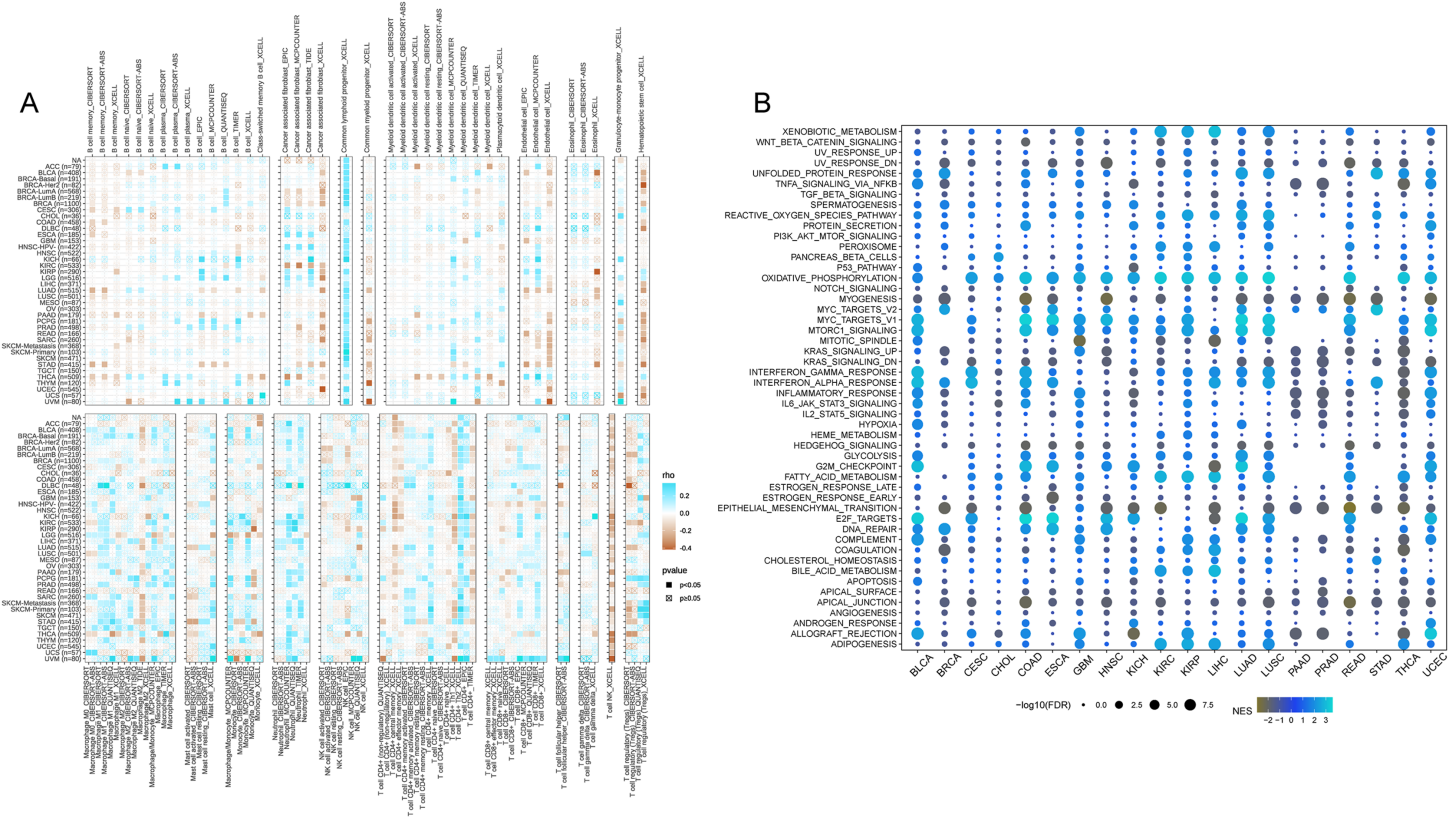
Pan-cancer immune inûltration enrichment analysis based on single genes. (A) Correlation between CISD2 and immune cells. (B) Investigation of CISD2 in tumors using Gene Set Enrichment Analysis (GSEA).

In accordance with the correlation analysis of CISD2 and immune cells, CISD2 exhibits a positive association with common lymphoid progenitors, granulocyte-monocyte progenitors, neutrophils, and CD8-positive T cells, providing an extensive insight into its influence on the tumor immune landscape. Nevertheless, a majority of tumors demonstrated negative correlations between CISD2 and endothelial cells, hematopoietic stem cells, and NK T cells. Patients with elevated CISD2 expression exhibited increased infiltration of tumor-eradicating CD8-positive T cells, leading to a more favorable prognosis (Fig. 8A).

#### Pan-cancer GSEA enrichment investigation

A comprehensive characterization of CISD2 in 20 malignancies revealed significant associations with cancer-related pathways (WNT-beta-catenin signaling, TNFA signaling through NFKB, TGF-beta signature, *etc*.), and oxidative metabolism-associated pathways (xenobiotic metabolism, reactive oxygen species pathway). However, a substantial correlation with epithelial cells was observed for CISD2 (Fig. 8B). A negative association between CISD2 and epithelial-mesenchymal transition, as well as apical junctions, may impede tumor proliferation and metastasis.

#### Ferroptosis cancer survival gene analysis

**Figure 9:**
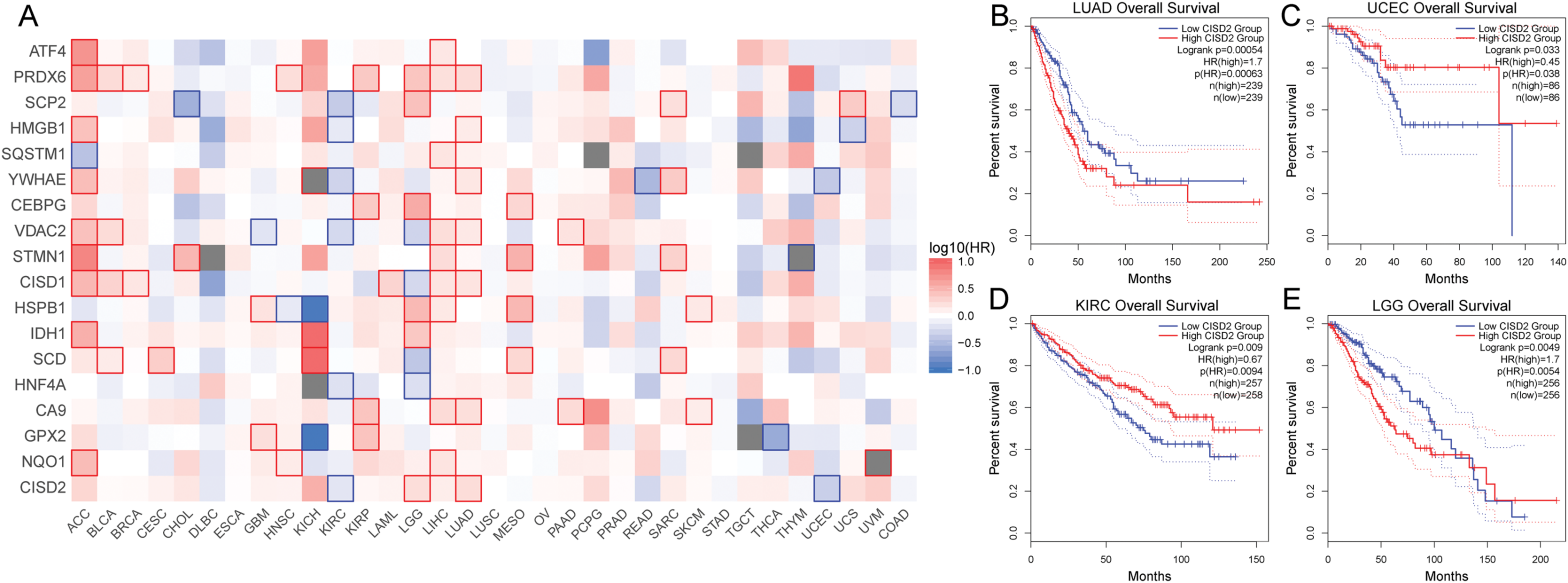
Pan-cancer survival analysis. (A) Survival outcomes of ferroptosis genes across various cancers. (B–E) Kaplan-Meier curves depicting CISD2 expression in lung adenocarcinoma (LUAD), uterine *corpus* endometrial carcinoma (UCEC), kidney renal clear cell carcinoma (KIRC), and lower grade glioma (LGG).

Subsequent survival analysis of the ferroptosis-colon cancer co-associated gene served to determine patient prognosis (Fig. 9A). Heterogeneity may exist among ferroptosis-related genes across tumor types. It was discerned that the prognostic impact of CISD2 varies considerably among distinct tumors based on median expression levels. In patients with LUAD and LGG, the prognosis was markedly superior in the low CISD2 expression cohort, whereas in patients with UCEC and KIRC, a significantly improved prognosis was observed in the high CISD2 expression cohort (Figs. 9B–9E). This study unveiled the overarching prognostic implications of ferroptosis in cancer.

## Discussion

Approximately two-thirds of patients with stage III colon cancer, along with certain individuals presenting high-risk stage II disease, undergo adjuvant chemotherapy to mitigate the recurrence risk following colectomy (Kannarkatt et al., 2017; Miller et al., 2022). By examining scRNA-seq samples from the GSE110009 dataset, we identified the crucial ferroptosis gene CISD2, enhancing patient survival outcomes.

CISD2, an enduring gene situated on human chromosome 4q, plays a significant role. Mice with Cisd2 knockout exhibit mitochondrial rupture, dysfunction, and autophagic cell death (Chen et al., 2009). Elevated CISD2 and CISD1 proteins have been observed in human epithelial breast cancer cells; inhibiting CISD2 and CISD1 expression results in increased iron accumulation within mitochondria, autophagy activation, and significant reduction of cell proliferation and tumor growth (Sohn et al., 2013). CISD2 modulates ER Ca^2+^ uptake and Ca^2+^ pump activity to maintain intracellular Ca^2+^ homeostasis through interactions with Serca2b. CISD2 deficiency disrupts calcium homeostasis, endoplasmic reticulum stress, NAFLD, and NASH. CISD2 hemizygous deletions have been identified in numerous human HCCs (Shen et al., 2017). Our analysis of CISD2 gene and copy number variation in colon cancer indicates that CISD2 significantly influences clinical prognosis and is more prone to undergo somatic copy number deletion.

In this investigation, CISD2 was determined to be essential for maintaining immune microenvironment homeostasis in colon cancer. CISD2 infiltration of T cells in colorectal cancer samples exhibited a positive correlation with CISD2 levels. According to the relationship between CISD2 and immune cells, colon cancer patients with high CISD2 expression and CD8+ T cells experienced improved outcomes. Furthermore, CISD2 expression was intimately linked to glioma progression and M2 polarization (Zhang et al., 2022). Consequently, CISD2 is hypothesized to impede tumor growth by promoting immune cell infiltration and preserving endoplasmic reticulum calcium homeostasis.

Moreover, we discerned an inverse correlation between the methylation status of CISD2 and two CpG sites within COAD specimens. It is well-established that aberrant DNA methylation, including hypermethylation of CpG islands in gene promoters, contributes to the silencing of tumor suppressor genes in cancer (Baylin, 2001; Esteller, 2002, 2007). This negative association suggests that heightened methylation of these specific CpG sites may lead to downregulation of CISD2 in COAD samples, potentially impacting the tumor microenvironment and patient prognosis. Nevertheless, further functional investigations are warranted to validate this hypothesis and elucidate the precise mechanisms involved.

Through single-cell and single-gene analyses of colon cancer tumor samples, we identified a correlation between CISD2 and clinical outcomes, with elevated CISD2 expression conferring a protective effect against colon cancer and suppressing the immune system. In this study, we meticulously examined the ramifications of differential CISD2 expression and genetic alterations on the clinical prognosis and associated mechanisms for colon cancer patients at both cellular and genetic levels, which is of paramount importance for investigating the iron regulatory mechanism in colon cancer. However, the specific mechanisms remain elusive, and there is a scarcity of clinical and experimental data. And potential confounding variables, such as patient age, gender, and tumor stage, may have influenced the observed associations between CISD2 expression and clinical outcomes. While we attempted to control for these variables in our analyses, residual confounding may still be present. Additionally, our findings are based on the analysis of existing databases and datasets, which may introduce biases related to data quality, sample selection, and study design. Future studies should incorporate independent cohorts and experimental validation to confirm our observations and further investigate the functional implications of CISD2 methylation and expression in COAD.

## Conclusion

By modulating the cell cycle and activating the immune system, CISD2 demonstrates statistical significance for prognosis in colon cancer. Consequently, CISD2 plays a crucial role in controlling the tumor microenvironment by regulating tumor homeostasis. The negative correlation between CISD2 methylation status and 2 CpG sites in COAD samples suggests a potential functional relevance in the downregulation of CISD2, but further studies are necessary to fully understand the underlying mechanisms and confirm these findings.

## Supplemental Information

10.7717/peerj.15476/supp-1 Supplemental Information 1 Data analysis and integration. (A) Consolidation of four single-cell samples from GSE110009; (B) Protein interaction networks for 18 genes; (C) The Cancer Genome Atlas (TCGA) and Genotype-Tissue Expression (GTEx) data representation before batch effect removal; (D) TCGA and GTEx data representation following batch effect removal; (E) Principal Component Analysis (PCA) validation of TCGA and GTEx data prior to batch effect elimination; (F) PCA validation of TCGA and GTEx data subsequent to batch effect elimination.

10.7717/peerj.15476/supp-2 Supplemental Information 2 Protein-protein interaction, enrichment analysis, and differential analysis. (A) Interconnectivity among CISD2 genes (B) Protein-Protein Interaction (PPI) illustrating the protein interaction network of CISD2 (C) Gene Set Variation Analysis (GSVA) enrichment analysis showcasing the disparity in enrichment between high and low CISD2 expression groups (D) GSEA enrichment analysis of CISD2-associated pathways (E) Pan-cancer comparison of CISD2 expression discrepancies between tumor and normal samples.

10.7717/peerj.15476/supp-3 Supplemental Information 3 Raw data from the cell experiment.

10.7717/peerj.15476/supp-4 Supplemental Information 4 Uncropped Blots of Fig. 7.

## Abbreviations

5-FU: 5-Fluorouracil
ANOVA: Analysis of Variance
BAP1: BRCA1-associated protein 1
CAFs: cancer-associated fibroblasts
CCK8: Cell Counting Kit-8
CCL5: chemokine (C-C motif) ligand 5
cDC1: conventional type 1 dendritic cells
CISD2: CDGSH Iron Sulfur Domain 2
CNV: Copy Number Variation
COAD: Colon Adenocarcinoma
DMSO: Dimethyl Sulfoxide
GEO: Gene Expression Omnibus
GO: Gene Ontology
GSEA: Gene Set Enrichment Analysis
GTEx: Genotype-Tissue Expression
KEGG: Kyoto Encyclopedia of Genes and Genomes
KIRC: Kidney Renal Clear Cell Carcinoma
LGG: Lower Grade Glioma
LUAD: Lung Adenocarcinoma

## Additional Information and Declarations

### Data Availability

The following information was supplied regarding data availability: The raw data are available in the Supplemental Files.
