# Supplementary figures and images for: Ferroptosis-associated gene CISD2 suppresses colon cancer development by regulating tumor immune microenvironment
Source: PeerJ. 2023 Jun 5;11:e15476. doi: 10.7717/peerj.15476 (PMC10249621; doi:10.7717/peerj.15476)

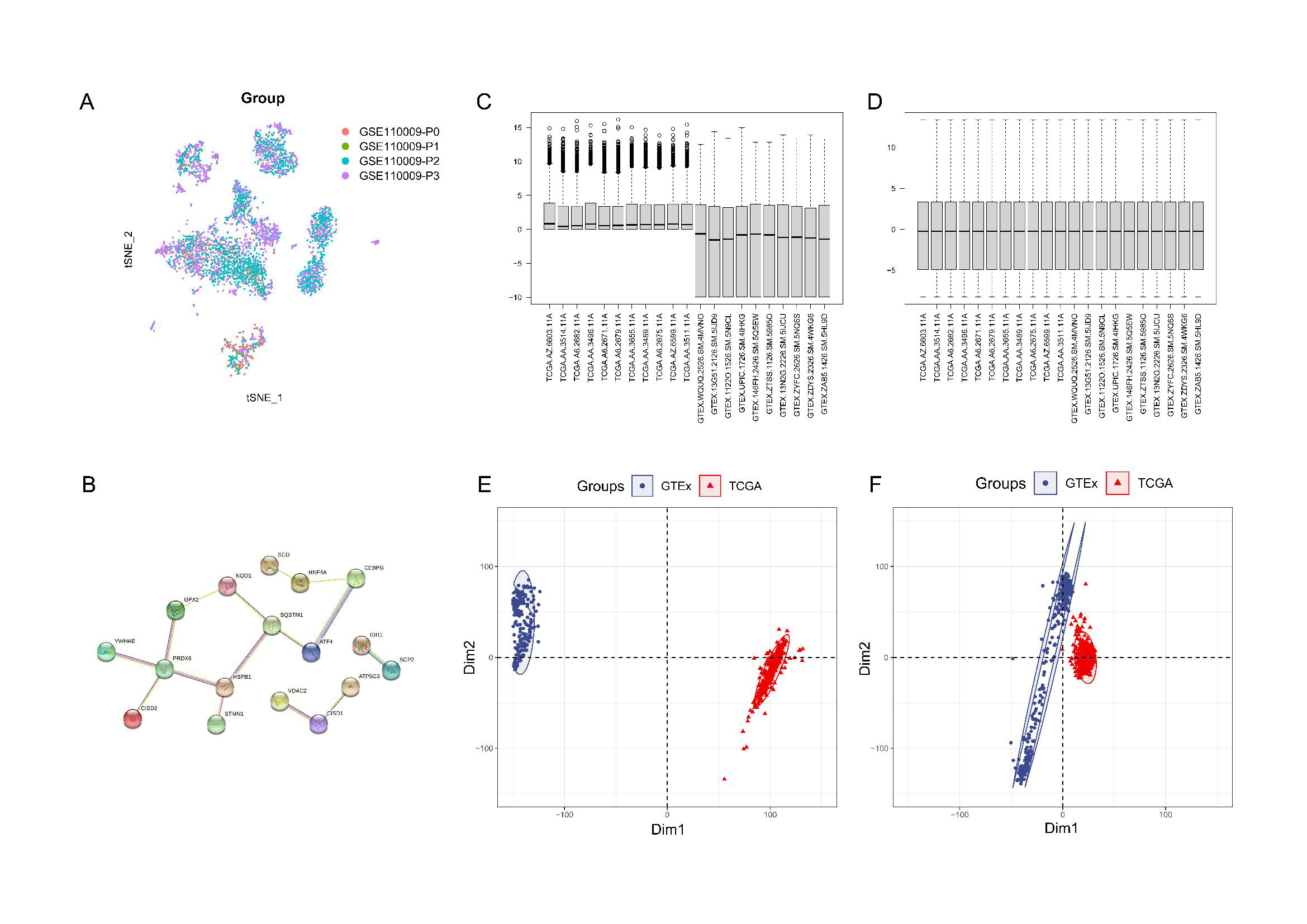

Supplement: Supplemental Information 1 — (A) Consolidation of four single-cell samples from GSE110009; (B) Protein interaction networks for 18 genes; (C) The Cancer Genome Atlas (TCGA) and Genotype-Tissue Expression (GTEx) data representation before batch effect removal; (D) TCGA and GTEx data representation following batch effect removal; (E) Principal Component Analysis (PCA) validation of TCGA and GTEx data prior to batch effect elimination; (F) PCA validation of TCGA and GTEx data subsequent to batch effect elimination. [file peerj-11-15476-s001.png]

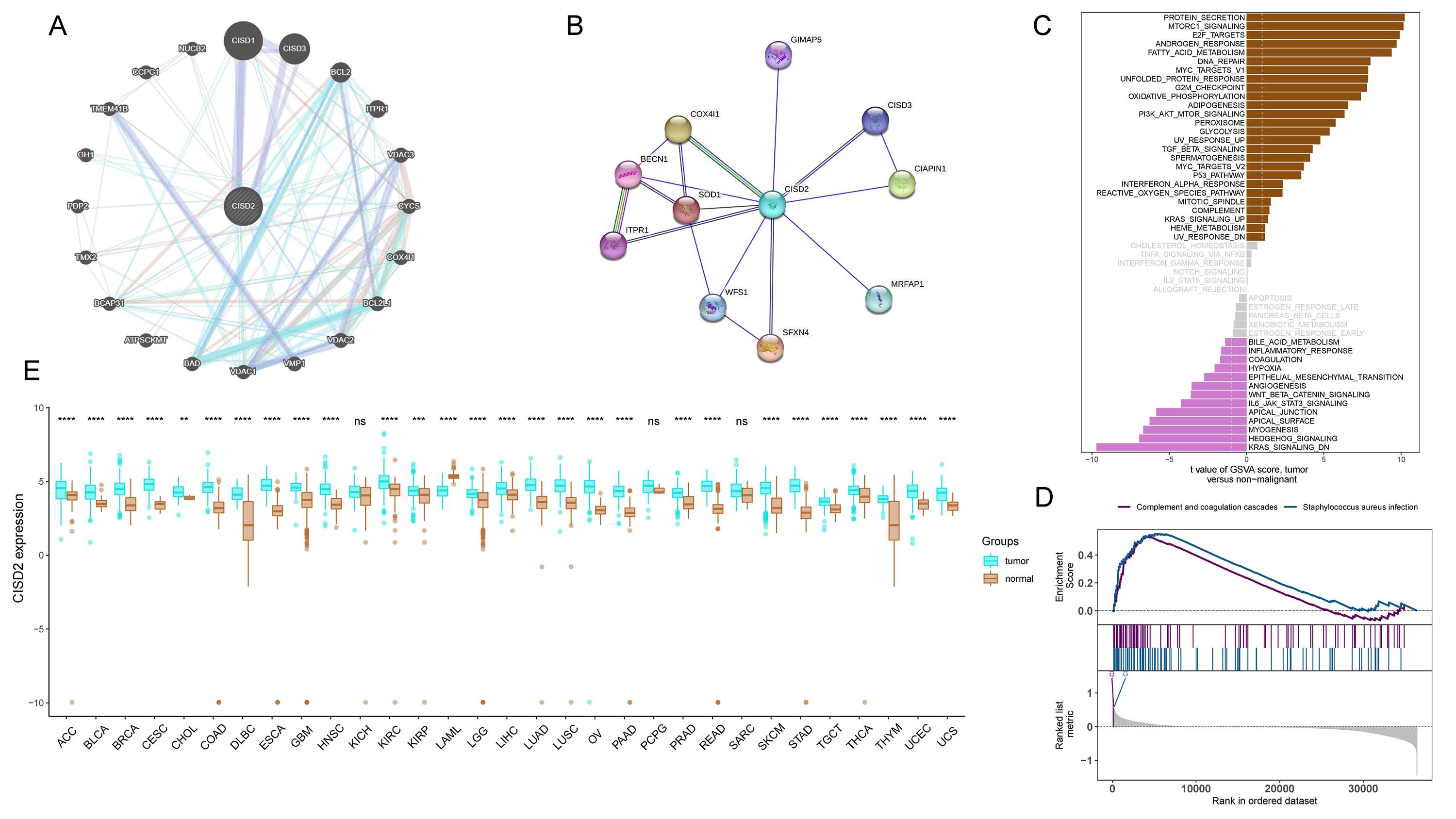

Supplement: Supplemental Information 2 — (A) Interconnectivity among CISD2 genes (B) Protein-Protein Interaction (PPI) illustrating the protein interaction network of CISD2 (C) Gene Set Variation Analysis (GSVA) enrichment analysis showcasing the disparity in enrichment between high and low CISD2 expression groups (D) GSEA enrichment analysis of CISD2-associated pathways (E) Pan-cancer comparison of CISD2 expression discrepancies between tumor and normal samples. [file peerj-11-15476-s002.png]

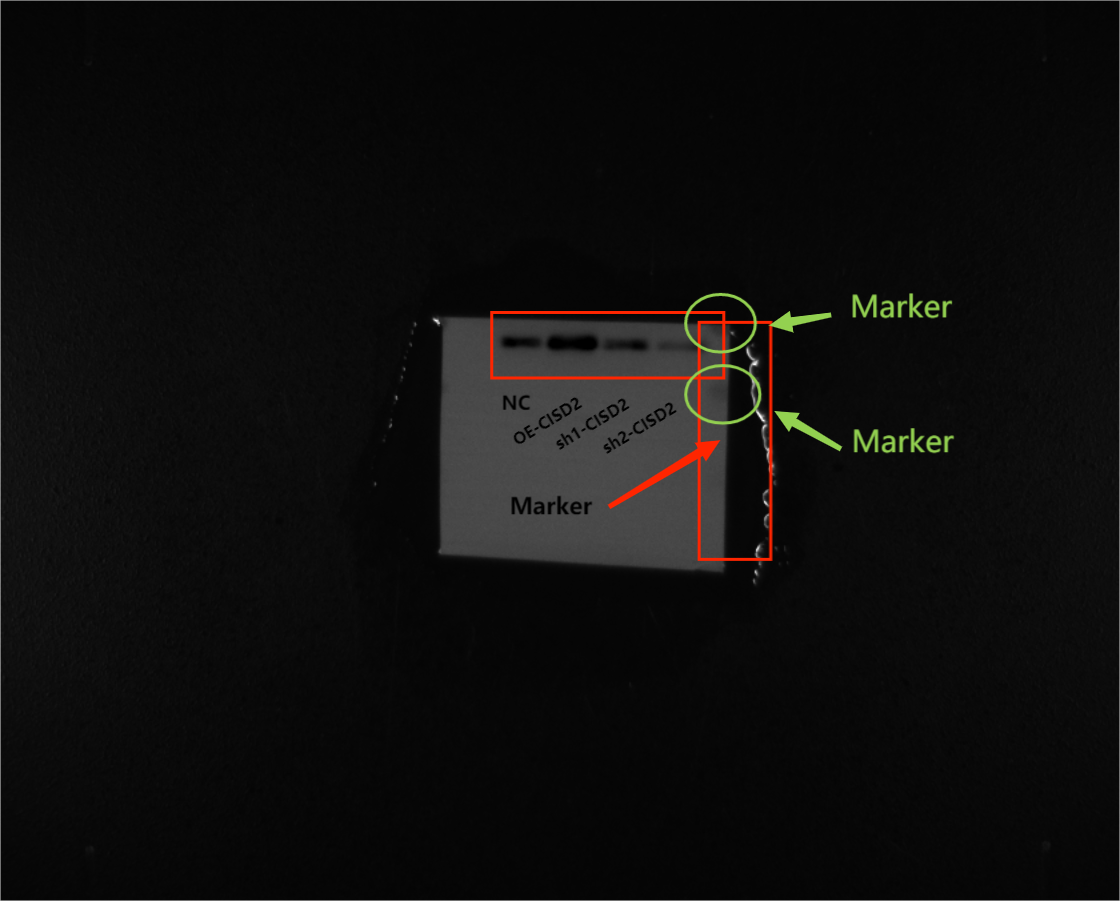

Supplement: Supplemental Information 4 [file peerj-11-15476-s004.zip › Uncropped Blots/CISD2 .png]

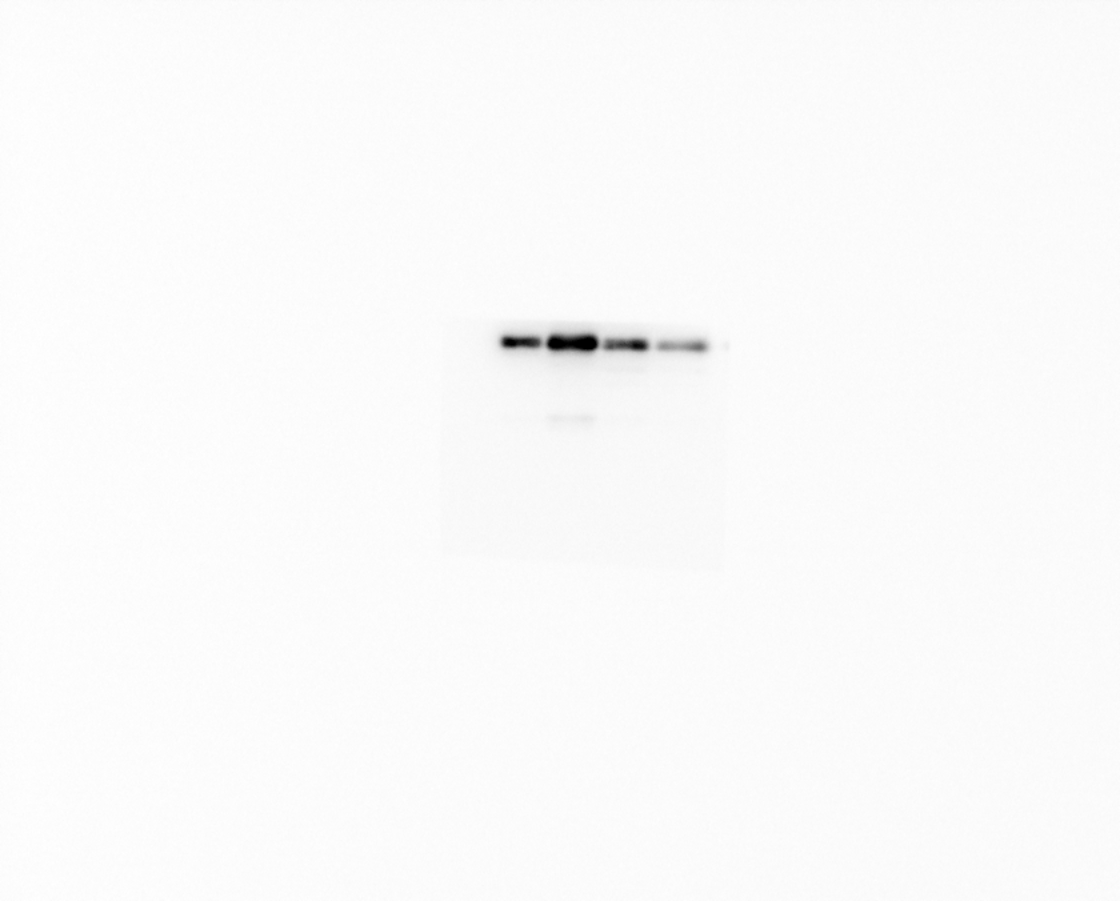

Supplement: Supplemental Information 4 [file peerj-11-15476-s004.zip › Uncropped Blots/CISD2 Sage_优化图.png]

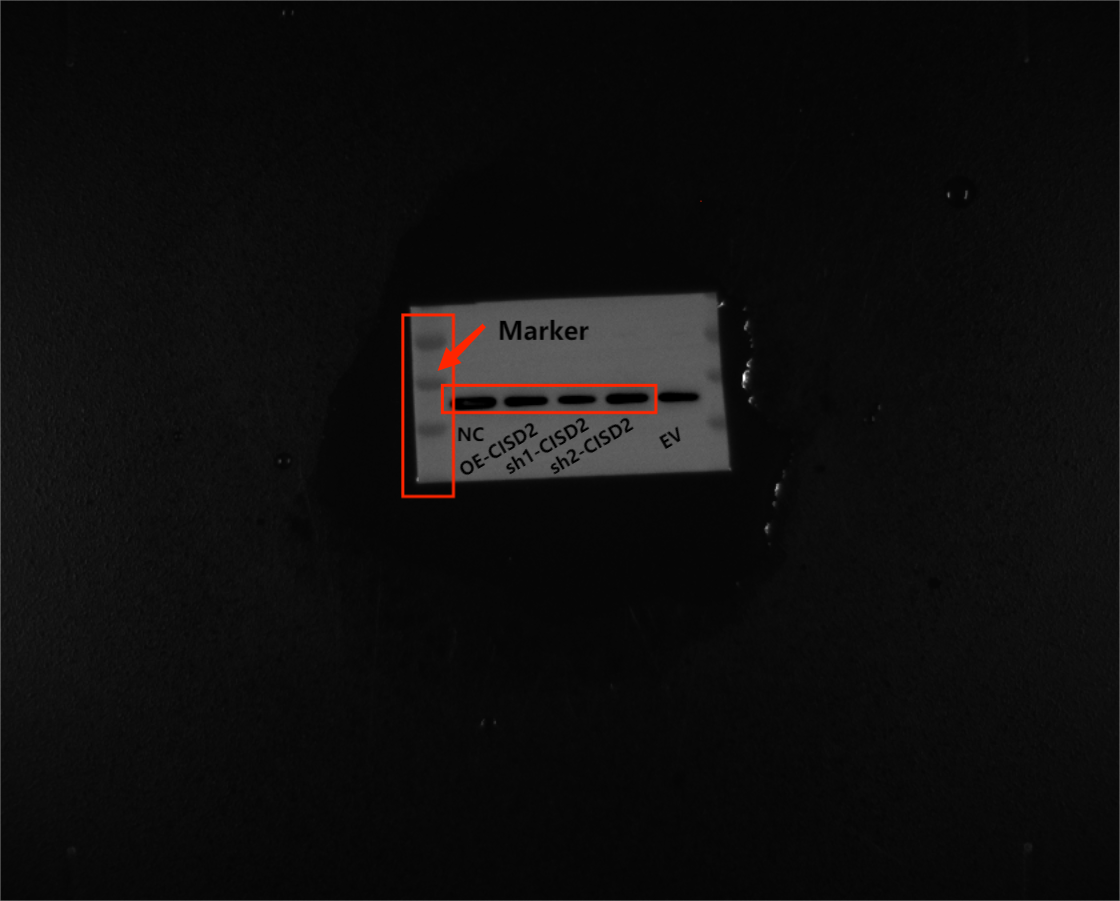

Supplement: Supplemental Information 4 [file peerj-11-15476-s004.zip › Uncropped Blots/GAPDH.png]

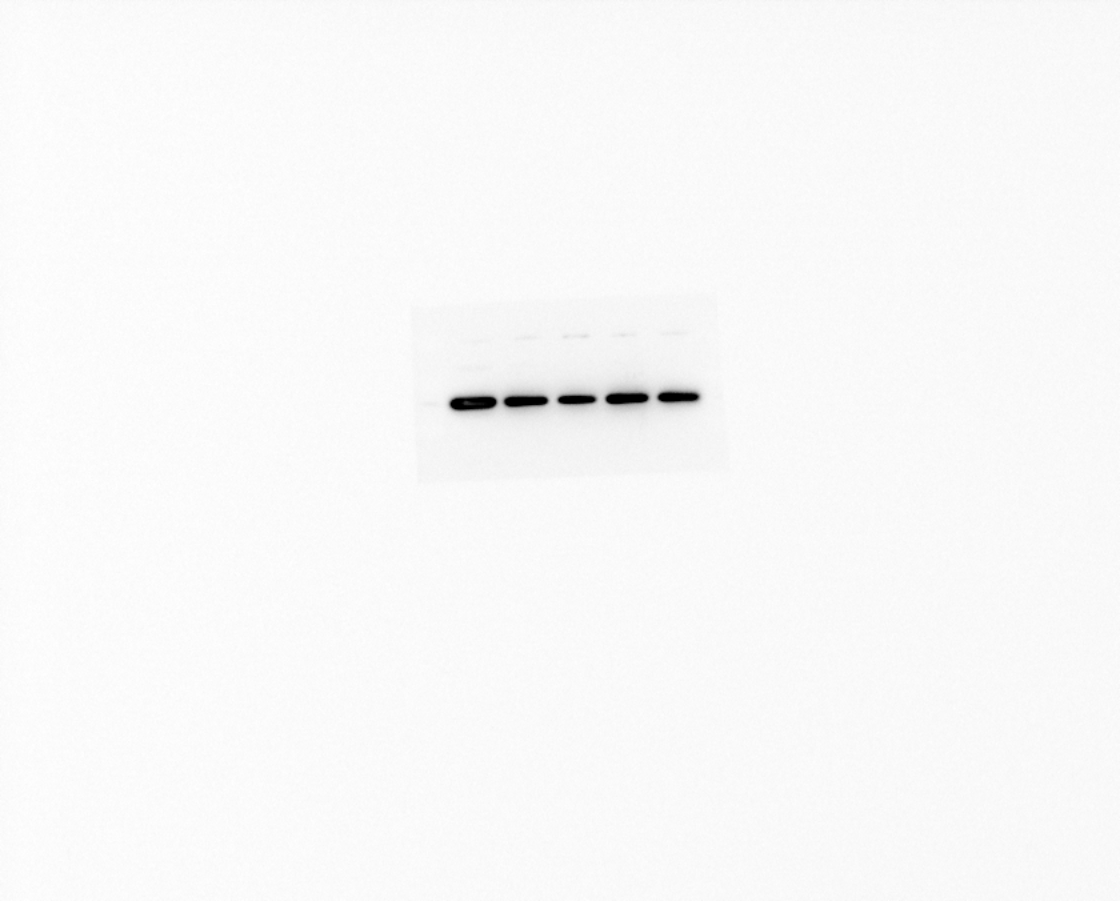

Supplement: Supplemental Information 4 [file peerj-11-15476-s004.zip › Uncropped Blots/GAPDH_优化图.png]
